# Interaction between the Circadian Clock and Regulators of Heat Stress Responses in Plants

**DOI:** 10.3390/genes11020156

**Published:** 2020-02-01

**Authors:** Tejasvinee Mody, Titouan Bonnot, Dawn H. Nagel

**Affiliations:** Department of Botany and Plant Sciences, University of California, Riverside, California 92507, CA, USA; tmody001@ucr.edu (T.M.); tbonnot@ucr.edu (T.B.)

**Keywords:** circadian clock, heat stress, thermotolerance, transcription factors, rhythmic gene expression

## Abstract

The circadian clock is found ubiquitously in nature, and helps organisms coordinate internal biological processes with environmental cues that inform the time of the day or year. Both temperature stress and the clock affect many important biological processes in plants. Specifically, clock-controlled gene regulation and growth are impacted by a compromised clock or heat stress. The interactions linking these two regulatory pathways include several rhythmic transcription factors that are important for coordinating the appropriate response to temperature stress. Here we review the current understanding of clock control of the regulators involved in heat stress responses in plants.

## 1. Introduction

The negative impacts on plant growth and development sustained from increasing environmental temperatures necessitate a comprehensive understanding of the underlying molecular and physiological interactions and mechanisms [1,2]. To adapt to changing environmental temperatures, plants take advantage of multiple regulatory and signaling pathways to optimize growth and fitness [3,4,5,6]. One such signaling pathway is known as the circadian clock—endogenous time-keeping machinery that accurately and precisely coordinates internal processes depending on the external surroundings [7,8,9,10,11,12]. The clock acts as a molecular switch, turning genes on or off when conditions are more or less favorable or when something requires an immediate response, primarily by regulating those genes’ transcription [13]. More broadly, the thermal effects on growth and fitness for some plants differ between day and night [14]. This is evident even in crop varieties with different sensitivity to heat stress, such as rice [15]. Therefore, insights into day versus night temperature stress responses, and more specifically time-of-day effects during the 24 h period, is critical for the accurate prediction of the appropriate molecular response to changing weather patterns for a variety of plants.

**Figure 1:**
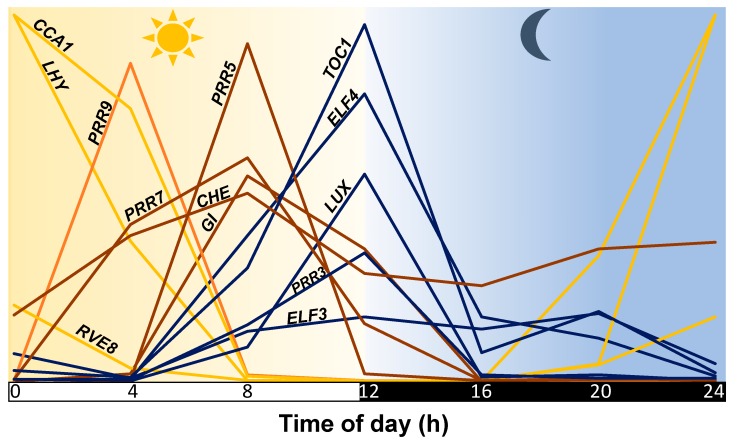
Timing of the peak expression of primary circadian clock components. Represented are normalized expression profiles obtained from DIURNAL, using experimental conditions LDHH_SM and LDHH_ST [29].

In *Arabidopsis,* where our understanding of the plant clock is most comprehensive, components of the oscillator interact at the transcriptional, post-transcriptional, and translational level to sustain robust ~24 h oscillations (rhythms) [16,17]. At the core are two MYB transcription factors (TFs), *CIRCADIAN CLOCK ASSOCIATED1 (CCA1)* and *LONG HYPOCOTYL (LH*Y), as well as a member of the pseudo-response regulators (PRRs), *TIMING OF CAB EXPRESSION1 (TOC1)* [18]. CCA1 and LHY show peak (highest) expression in the morning and repress *TOC1*; thus, *TOC1* expression is partitioned to the evening [19]. TOC1 in turn regulates the expression of *CCA1* and *LHY*, forming the core clock feedback loop [20,21]. Throughout the day/night cycle, the core loop regulates and contributes to the timing of peak expression for other primary clock components (Figure 1) [22,23,24,25,26,27,28]. For example, in the morning and during the day, additional loops composed of CCA1, LHY, PRR7, and PRR9, and in the evening, components of the evening complex (EC), all play critical roles in sustaining normal clock function [25,26,27].

Coordination between clock components and environmental signals, such as light and temperature, contribute to clock robustness and optimal plant growth [8,9,10,30,31,32,33]. In *Arabidopsis*, a greater understanding of the interaction between light signals and the clock is known [33]. However, the relationship status between temperature and the clock is complicated. Within the range of physiologically relevant temperatures (~12- 32 °C), the clock can be entrained by cycles of hot and cold, but will buffer against prolonged temperature changes to maintain a ~24 h periodicity, referred to as compensation [30,34,35,36]. CCA1, LHY, PRR7, and PRR9, along with a few other components, have been implicated in the regulation of entrainment and compensation depending, on the temperature range [11,24,36,37]. In addition, TOC1 and the photoreceptor phytochrome B (PHYB) have also been linked to high ambient (warm) temperature signaling in the clock [38,39,40]. Together, warm temperatures impact multiple levels of gene regulation, including alternative splicing, post-translation, and protein abundance [12,34,41,42]. More recently, it has been shown that the impact on the transcript abundance of clock genes following a 1 h exposure to 30 °C differs depending on the time of day [43]. Circadian gating (controlling the magnitude or the occurrence of a response based on time of day) of cold responses for clock and clock-controlled genes have been previously reported [44]. Furthermore, the cold induction of several TFs is dependent on time of day [44,45,46]. Therefore, crosstalk between heat- and cold-gated responses is likely to intersect for several clock and clock controlled genes. Interestingly, the binding of CCA1 to clock targets is enhanced at a high ambient temperature (~30 °C) while the opposite effect is observed for the evening clock components association with their targets [47,48]. Members of the heat shock protein family (HSP90.2) also contribute to the adjustment of the circadian period in warm–cold-entrained seedlings [49]. However, the precise mechanisms of how the clock maintains this remarkable balance between sensitivity and resistance to changes in ambient temperatures at the molecular level remains poorly understood in plants and many other organisms. Several insightful reviews have been written on how high ambient temperature interacts with the clock in plants [6,11,12,30]. This review primarily focuses on how temperatures outside the range of compensation (heat stress) impact the expression of clock genes and clock-controlled TFs.

## 2. Regulation of Clock Genes by Heat Stress in Plants

Outside the range of compensation, exposure to extreme temperatures alters clock gene expression, and thus clock-controlled processes [50,51,52,53]. Up to 50% of the genes responsive to temperature stress show diurnal or circadian oscillations in *Arabidopsis* [53]. It is therefore not surprising that together, heat stress and the clock significantly influence how plants interact with a changing environment. Mutants for several clock components impact the plant’s ability to tolerate and acclimate to temperature stress [13,54,55,56,57,58]. In addition, recent studies indicate that the F-box protein ZEITLUPE (ZTL) and HSP90.2 are required for proper clock function under heat stress, as they modulate protein quality control [51].

**Figure 2:**
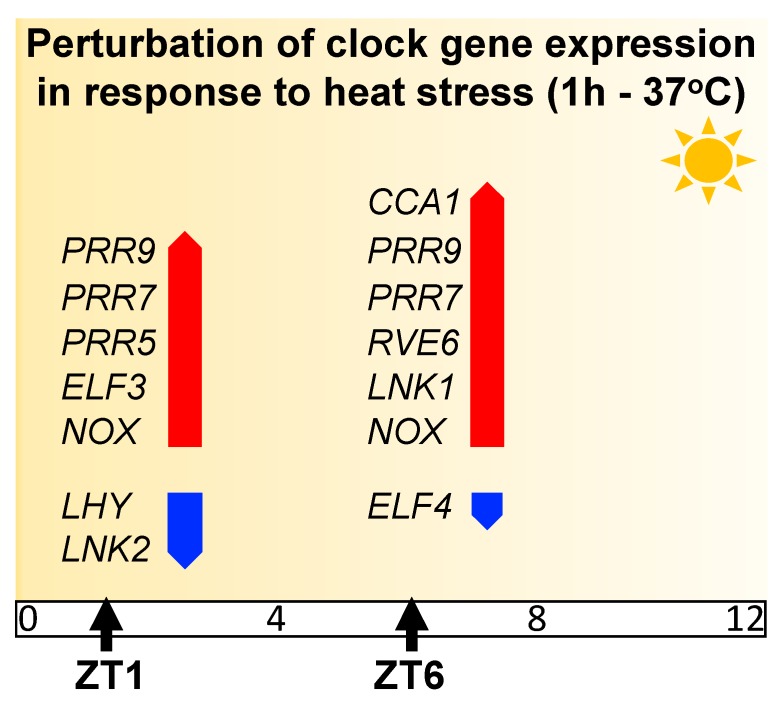
Impact of heat stress (37 °C) on transcript abundance of *Arabidopsis* clock components at two different times of the day (morning (ZT1) or afternoon (ZT6)). Upregulation (red arrow) and downregulation (blue arrow) are based on log2 fold change values obtained from RNA-seq analysis [50]. Only genes with statistically significant (FDR < 0.05) differences are represented.

Emerging transcriptome studies suggest that during the day, the plant transcriptional response to heat stress is quite dynamic [50,59]. In *Arabidopsis*, the transcript abundance of clock genes is altered in response to heat stress depending on the time of day [50] (Figure 2). *CCA1, PRR7, PRR9,* and other primary components show increased mRNA abundance to heat stress, with greater induction occurring at specific times of the day (morning (ZT1) or afternoon (ZT6)). In contrast, *LHY*, which is often considered to act redundantly with *CCA1*, shows reduced transcript abundance under heat stress, suggesting that in response to specific environmental stimuli, they might have distinct functions [50] (Figure 2). 

Outside of *Arabidopsis*, clock genes are found across angiosperm genomes [60]. For the most part, the rhythmic time-of-day expression of clock and clock-controlled genes in crop species appears to be conserved [61,62,63,64]. For example, in monocots, such as rice and maize, the circadian phase of the core clock genes, *CCA1* and *TOC1,* display similar time-of-day expression and flowering phenotypes, as observed in *Arabidopsis* [65,66,67]. Based on the available expression data in Genevestigator for crops, the transcript abundance of clock components appears to be impacted by heat stress to varying degrees [68]. Furthermore, in soybean, the perturbation of primary clock components in response to heat stress alters normal clock function [69]. Although global studies on the clock control of heat stress-responsive genes in many crops are not available, genes responsive to other abiotic stress show rhythmic expression in barley and soybean, suggesting that temperature stress-responsive genes might also be regulated by the clock in these species, and thus in crops in general [70,71]. In crassulacean acid metabolism (CAM) plants, which are known to be able to tolerate high temperatures and arid habitats, the clock also appears to be driven by a multigene oscillator [72,73,74]. In *M. crystallinum*, the adjustment of the timing of peak expression for some clock genes in response to a temperature pulse during the night might occur [72,75]. Furthermore, in pineapple, the promoters of CAM photosynthesis genes are enriched for *cis*-elements bound by clock genes [76]. However, the expression of *Opuntia ficus-indica* (cactus) clock genes *OfiCCA1* and *OfiPRR9* oscillate with a 12 h periodicity, unlike Arabidopsis, suggesting that the circadian clock in some CAM plants may be different from C3 and C4 plants [77]. Whether heat stress impacts the expression of clock and clock-controlled genes in a time-dependent context in CAM and C4 plants remains to be determined.

## 3. Time-of-Day Regulation of Heat Shock Transcription Factors in Plants 

**Figure 3:**
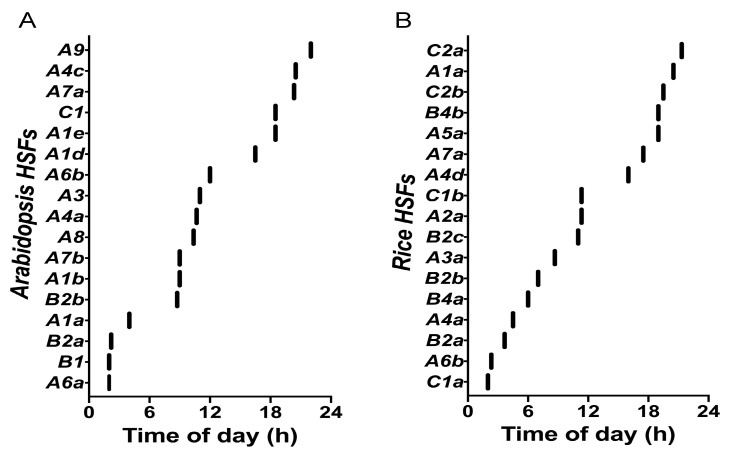
Timing of peak expression (phase) for plant heat shock transcription factors (*HSFs*) in (**A**) *Arabidopsis (AtHSFs)* and (**B**) rice *(OsHSFs)*. The phase data was obtained from DIURNAL, and the average phase was calculated across all conditions available in DIURNAL for each plant species [29].

Downstream of the clock network, the expression of multiple genes is clock-regulated, in order to provide the optimal response to stress depending on the time of day. In plants, exposure to elevated temperatures activates the heat shock response (HSR) pathway and primarily triggers the synthesis of heat shock proteins (HSPs) [78,79,80]. The induction of HSP biosynthesis is, however, regulated by heat shock TFs (HSFs) [80,81]. *Arabidopsis* contains 21 *HSFs,* and interestingly, the transcript abundance of 17 of these *HSFs* show rhythmic expression [29]. Five *HSFs* cycle under free-running (constant) conditions, whereas 16 *HSFs* show rhythmic expression under photocycles or thermocycles [29]. Together, *HSFs* are expressed throughout the day (morning, afternoon, and evening), with at least one *HSF* showing timing of peak expression (phase) every 4 h (Figure 3) [29,81]. 

Although not all *HSFs* respond to heat stress, this TF family plays a role in other abiotic stress responses [50,59,81,82,83]. The observation that they are available at almost all time points during the day supports an important functional role of HSFs in the plant response to external stress signals, which is most likely mediated by the clock. *HSFA1e, A3, A4a, A6b, A8, B2aI,* and *C1* are direct targets of clock genes based on chromatin immunoprecipitation followed by deep sequencing (ChIP-seq); however, experimental evidence to link the biological significance of these interactions needs to be further examined [84,85,86,87,88]. Interestingly, one of the *HSFs (HSFB2b)* has been shown to play a role in the clock’s ability to buffer against certain temperature changes, suggesting a feedback regulatory relationship between the time, temperature, and *HSFs* [89]. This interaction between the clock, heat stress and HSFs can be discerned from RNA-seq datasets that reveal how the time of day regulates *HSFs’* induction in response to heat stress. In *Arabidopsis, HSFA2* and *HSFA7b* are induced by heat stress in the morning (ZT1) and afternoon (ZT6), respectively, and the induction is higher in the afternoon [50]. In contrast, *HSFA3* transcript abundance accumulates to higher levels in the afternoon relative to the morning [43,50]. *HSFB1*, *HSFB2a,* and *HSFB2b* are all heat-induced with higher transcript abundance in the afternoon, whereas *HSFC1* gene expression is repressed by heat regardless of time of day [50,90]. 

In rice, of the 25 *HSFs* that are present, 17 show rhythmic expression under diurnal conditions (light or temperature); interestingly, 11 out of those 17 are induced by heat stress (Figure 3) [29,91]. For the most part, the time of peak expression of cycling *HSFs* is not consistently conserved between *Arabidopsis* and rice. However, a few *HSFs* belonging to either the *HSF* A, B, or C clade appear to show highest expression at similar phases of the day for both species. For example, *AtHSFA7a*, *AtHSFC1*, *OsHSFA7a, OsHSFC2a*, and *OsHSFC2b* show peak expression in the early morning (ZT18–24); *AtHSFB2a* and *OsHSFB2a* peak in the morning (ZT0–ZT4); *AtHSFB2b* and *OsHSFB2b* peak in the afternoon to early evening (ZT6–ZT8); and *AtHSFA3* and *OsHSFA3a* peak in the evening (ZT8–ZT12) (Figure 3). Furthermore, other *HSFs* are also expressed at different times of the day. Taken together, *HSFs* in *Arabidopsi*s and rice are expressed throughout the day, but each individual heat-responsive *HSF* cycles with a unique phase of peak expression during the 24 h period, suggesting temporal partitioning of gene expression in response to temperature stress (Figure 3). Interestingly, in pineapple, five HSFs are regulated by the circadian clock and show peak expression in the morning [92]. This suggests that even CAM plants that might be more tolerant to extreme temperature environments, and that heat responsive regulators are controlled in a time-of-day-dependent thermotolerance mechanism. 

While HSFA1s are the master transcriptional regulators of the early heat stress response in plants, the clock genes *REVEILLE4 (RVE4)* and *RVE8* might play key roles in an HSF-independent pathway during the first wave of the heat stress response [59]. The role of *RVE8* in plant thermotolerance is gated by the circadian clock, with a specific regulation around ZT6 to ZT7 [59]. However, the highest thermotolerance occurs at ZT15 to ZT16, suggesting the activation of additional clock-controlled regulators at this particular time of day [59]. 

Since plants are subjected to varying temperatures throughout the day, with the highest temperature experienced around or after noon, their physiological response to temperature also differs throughout the 24-hour day. Hence, plants need to have time-of-day-dependent temperature response, which at the cellular level requires gene regulation by multiple TF families. 

Thus, further insights into the influence of the time of day on heat stress events for cycling transcriptional networks will help to decipher the dynamic nature of the heat stress response throughout the day. 

## 4. Time of Day and other Clock-Regulated Heat Responsive Transcription Factor Families

In response to abiotic stresses, several TFs act as master regulators of the responsive pathways. TFs exhibiting circadian oscillations and directly targeted by clock genes could have a specific role during the day in response to a particular stress. From the list of differentially expressed genes identified in two recent heat responsive transcriptomes, ~12% and ~9% of the upregulated and downregulated transcripts, respectively, correspond to TFs [50,59,93] (Table S1). Of these, ~50% (308 TFs) exhibit circadian oscillations in constant light conditions, and are members of 59 different families, highlighting the diversity of the transcriptional regulators controlled by the circadian clock and involved in the heat stress response [29]. An in-house enrichment analysis highlighted 17 families that are over-represented in at least one heat responsive transcriptome dataset, compared to all *Arabidopsis* TFs, which suggests that these TF families might be important in context-dependent regulatory hubs (Table S1). The *GRAS* and *MYB* TF families are two of the most over-represented families in cycling transcripts that are downregulated in response to heat, especially during the early response (Table S1). These two families participate in many aspects of plant growth and development [94,95]. Thus, transcriptional regulation of these family members under heat reflects the adjustment of developmental processes in response to this stress. For example, *MYB59* expression peaks in the afternoon, and its transcript abundance has been shown to be reduced within 10 min following exposure to heat, but only between ZT3–ZT6 [59]. Similarly, the *GRAS* member *SCL13* is upregulated after 1 h of heat stress exposure but only in the early morning, when its expression starts to increase [50,96,97]. Interestingly, overexpression of *OsMYB55* results in improved plant growth and performance under high temperature in both rice and maize [98,99]. The *Arabidopsi*s *MYB59* and *SCL13* TFs not only oscillate in constant light conditions, but are also direct targets of clock genes, suggesting that they might be key proteins mediating crosstalk between the clock and the plant response to elevated temperature [84,85,87,100].

In response to abiotic stresses, the large *AP2-EREPB* family plays an important role, and is well represented in heat-inducible TFs that exhibit circadian oscillations (Table S1). *DREB2A* is expressed in the evening, and functions as a key regulator of drought tolerance [101,102]. DREB2A activates the expression of *HSFA3*, which contributes to the establishment of thermotolerance [101,103]. Upregulation of *DREB2A* is detected after 15 min of heat exposure, followed by the upregulation of *HSFA3* after 30 min, and high transcription levels for both genes are maintained after 1 h, regardless of time of day [50,59]. A similar response has been observed for *DREB2B,* and overexpression of the rice *OsDREB2A* and *OsDREB2B* showed improved plant tolerance to drought, suggesting a conservation of the regulatory mechanisms across plant species [104,105]. Other *DREB* TFs, designated *CBFs*, have been shown to be regulated by cold, high ambient temperatures, and heat stress, implicating them as important regulators linking the clock to temperature stress response mechanisms [43,44,50,58].

Cycling *bZIPs* are also well-represented in heat responsive TFs (Supplementary Table S1), and crops over-expressing bZIP proteins have been reported to better tolerate abiotic stresses [106,107,108]. In addition, high ambient temperatures revealed the enrichment of a *bZIP* TF binding element in night-responsive genes [43]. The morning-expressed *ABF3* is upregulated in response to heat stress at ZT6 [50]. ABF3 is a master regulator of *ABA-*regulated genes, and its overexpression improved plant tolerance to drought stress in rice [109]. Because drought often occurs in warm conditions, many TFs regulated by heat also act in drought responsive pathways. Thus, modulation of the expression of many of these TFs might result from the coordination of multiple pathways commonly activated in abiotic stress responses (e.g., temperature, drought, hormone and ROS signaling, calcium-mediated stress signaling, redox homeostasis, etc.).

Although we have discussed the major TF families above, other oscillating TFs play important roles in thermotolerance, and are not necessarily over-represented in the heat responsive transcriptomes assayed in this review. For example, several members of the *bHLH* family, such as *PHYTOCHROME-INTERACTING FACTORS (PIFs)*, have been identified in heat-induced transcriptomes. *PIF4* has been shown to be upregulated in response to hot and warm temperatures in the early morning and at night, respectively [43,50]. Moreover, transcriptomic and ChIP-seq analyses also revealed *PIF1* and *PIF5* as cycling TFs, directly bound by clock genes and upregulated in response to warm temperature [20,29,43,84,86,88,110]. *MBF1C* is a central regulator of plant thermotolerance [111]. Under heat, its transcript abundance increases after 5 min, and MBF1c regulates the expression of *DREB2A* and two *HSFBs*, demonstrating its role in the interplay between the clock and the HSR pathway [59,112]. Together, these collective connections highlight the multi-level, time-of-day TF network underlying heat stress responses in plants.

## 5. Integrated Time of day and Heat Stress Transcriptional Networks

**Figure 4:**
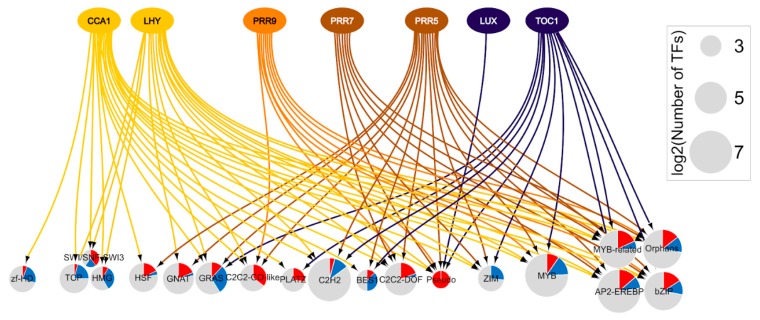
Transcriptional network of heat-responsive transcription factor families that are also cycling. The represented TF families are significantly over-represented in these high-temperature-responsive transcriptome datasets [43,50,59] (Supplementary Table S1), and include direct targets of CCA1, LHY, PRR7, PRR9, PRR5, LUX, and TOC1, based on ChIP-seq datasets [20,84,85,86,87,88,100,110]. The arrows in the network represent an interaction between the TF and its target. These connections indicate that at least one member of the TF family is a target of clock genes; however, not all members of the TF family are responding to heat. The node size for TF families reflects the size of the family, with the Log2-transformed total number of TFs. The colors in pie charts represent the proportion of TFs differentially expressed in response to heat. Blue pie: downregulated; red pie: upregulated; grey pie: not significant.

From transcriptomic datasets generated in Blair et al. [50], Grinevich et al. [43], and Li et al. [59], 169 TFs responding to hot or warm temperatures cycle under constant light and are direct targets of the clock [20,29,84,85,86,87,88,100,110]. These TFs are members of 19 TF families over-represented in differentially expressed genes (Figure 4). CCA1 and LHY target most of the represented families, highlighting their importance in regulating heat responsive TFs (Figure 4). Because most of the ChIP-seq data were not obtained at high temperatures, it would be interesting to study the influence of heat stress on the target selection of output genes. Nonetheless, using such network representation allows us to identify the specificity of clock genes for a particular heat-responsive TF family, and to visualize TF families that are targeted by one or multiple clock genes. Interestingly, the PRR family is the only represented family that is targeted by all the analyzed clock genes (Figure 4). All five members of this family were upregulated in response to heat (*PRR1, PRR3, PRR5, PRR7*, and *PRR9*), and are targets of at least one clock gene. (Figure 4).

To identify the regulators of plant thermo-responses, comparing heat-responsive transcriptomes is often complicated by the use of different experimental conditions, materials (temperature, light intensity, plant age, etc.), and data analysis methods (pipelines used for RNA-Seq data analysis, statistical models, and thresholds used to identify differentially expressed genes, etc.). However, despite the disparities between the methods, the differences of differentially expressed TFs between studies also demonstrate the specific role of the heat stress-responsive TFs. This specificity probably reflects the influence of the time of day, the duration of exposure, and the temperature applied to the plants. This also emphasizes the importance of combining data to obtain the most representative vision of the regulatory mechanisms involved in the plant response to stresses in a clock-dependent context.

## 6. Future Perspective

Major advances in the development and improvement of food crops is absolutely essential for feeding and nourishing the increasing world population [113]. In order to understand how plants respond to extreme environmental stress, comprehensive knowledge of the genes, regulatory pathways, and underlying mechanisms are critical [1,3,5,114]. Emerging studies reinforce that plant growth and survival under heat stress is directly dependent on the time of day, and that the clock integrates these signals to modulate timed responses that likely contribute to enhancing plant fitness. In this review, we mainly focus on components of the circadian oscillator and downstream TFs that respond to heat stress. Future gene-editing techniques to control and fine-tune the response to temperature stress by modifying promoters of heat-responsive and clock-controlled genes will be invaluable. In addition, a mechanistic understanding of how heat stress impacts all levels of gene regulation and how genes interact to promote a singular response to heat stress vs. a general stress response is needed. Modeling and network analyses that integrate multiple levels of gene regulatory networks will no doubt shed light on how key biological events will be perceived and processed in plants in future environmental changes. Integrated knowledge will enable the generation of predictive models for outcomes of plant tolerance in the context of climate change.

## Supplementary Materials

The following are available online at https://www.mdpi.com/2073-4425/11/2/156/s1, Table S1. Enrichment of rhythmic transcription factor families in heat responsive transcriptomes.
