# Supplementary material for: Interaction between the Circadian Clock and Regulators of Heat Stress Responses in Plants
Source: Genes (Basel). 2020 Feb 1;11(2):156. doi: 10.3390/genes11020156 (PMC7074488; doi:10.3390/genes11020156)
Supplement: Supplementary file 1 [file genes-11-00156-s001.pdf]

Table 1. Enrichment of rhythmic transcription factor families in heat responsive transcriptomes. TF family enrichment was calculated from two recent heat responsive transcriptome datasets (Blair *et al.*, 2019 and Li *et al.*, 2019), restricted to TFs exhibiting circadian oscillations under constant light condition, based on data from the DIURNAL project (Mockler *et al.*, 2007) and from the list of TFs reported in *Arabidopsis* (Pruneda-Paz *et al.*, 2014). Families with at least two members in at least one study are represented. Numbers of TFs, fold enrichments and *P*-values (Fisher's exact test) for each TF family and for each study are indicated in columns 'N', 'Fold' and '*P*', respectively. Total numbers of *Arabidopsis* TFs are indicated in parenthesis for each family in the first column. Colors indicate a significant difference ($P < 0.05$) of enrichment in the corresponding dataset compared to all *Arabidopsis* TFs, green indicating an over-representation of the family, orange indicating an under-representation.

TF Family (number of TFs)	**Blair *et al.* (2019)**						**Li *et al.* (2019)**					
	**Down-regulated TFs**			**Up-regulated TFs**			**Down-regulated TFs**			**Up-regulated TFs**		
	**N**	**Fold**	***P***	**N**	**Fold**	***P***	**N**	**Fold**	***P***	**N**	**Fold**	***P***
ABI3-VP1 (61)	0	0.0	1.1E-01	1	0.3	3.7E-01	0	0.0	2.6E-01	2	1.5	4.0E-01
AP2-EREBP (146)	3	0.5	2.1E-01	10	1.3	4.5E-01	3	0.6	4.8E-01	10	3.1	1.5E-03
BES1 (8)	3	8.5	9.5E-03	0	0.0	1.0E+00	1	3.7	2.6E-01	0	0.0	1.0E+00
bHLH (149)	8	1.2	5.4E-01	3	0.4	8.5E-02	7	1.4	3.5E-01	2	0.6	7.7E-01
bZIP (75)	3	0.9	1.0E+00	10	2.5	1.0E-02	4	1.6	3.3E-01	4	2.4	8.9E-02
C2C2-CO-like (17)	0	0.0	1.0E+00	4	4.4	2.0E-02	0	0.0	1.0E+00	3	8.0	8.4E-03
C2C2-DOF (36)	1	0.6	1.0E+00	4	2.1	1.4E-01	0	0.0	6.3E-01	2	2.5	2.0E-01
C2C2-GATA (30)	1	0.8	1.0E+00	1	0.6	1.0E+00	0	0.0	6.2E-01	1	1.5	4.9E-01
C2H2 (148)	12	1.8	4.2E-02	4	0.5	1.8E-01	6	1.2	6.4E-01	1	0.3	3.7E-01
C3H (69)	3	1.0	1.0E+00	1	0.3	2.6E-01	0	0.0	1.7E-01	0	0.0	4.0E-01
CCAAT (43)	1	0.5	1.0E+00	4	1.7	3.0E-01	0	0.0	4.0E-01	2	2.1	2.5E-01
FHA (17)	2	2.7	1.9E-01	0	0.0	1.0E+00	0	0.0	1.0E+00	0	0.0	1.0E+00
G2-like (40)	1	0.6	1.0E+00	4	1.9	2.8E-01	2	1.5	4.0E-01	1	1.1	5.9E-01
GNAT (32)	1	0.7	1.0E+00	2	1.2	6.9E-01	1	0.9	1.0E+00	1	1.4	5.2E-01
GRAS (34)	7	4.7	1.4E-03	3	1.6	4.3E-01	6	5.2	1.6E-03	1	1.3	5.4E-01
HB (91)	5	1.2	6.0E-01	4	0.8	1.0E+00	4	1.3	5.5E-01	1	0.5	7.2E-01
HMG (12)	3	5.7	2.3E-02	1	1.5	4.9E-01	0	0.0	1.0E+00	0	0.0	1.0E+00
HSF (23)	1	1.0	1.0E+00	3	2.4	1.4E-01	0	0.0	1.0E+00	3	5.9	1.7E-02
mTERF (34)	2	1.3	6.6E-01	3	1.6	4.3E-01	5	4.4	7.9E-03	0	0.0	1.0E+00
MYB (147)	6	0.9	1.0E+00	10	1.3	4.5E-01	12	2.4	4.7E-03	3	0.9	1.0E+00
MYB-related (72)	4	1.3	5.6E-01	8	2.1	6.3E-02	0	0.0	1.7E-01	1	0.6	1.0E+00
NAC (110)	1	0.2	8.8E-02	10	1.7	1.3E-01	5	1.3	4.2E-01	5	2.1	1.0E-01
ND (145)	7	1.1	8.3E-01	5	0.6	4.4E-01	3	0.6	6.3E-01	0	0.0	7.3E-02
Orphans (83)	4	1.1	7.8E-01	10	2.2	2.5E-02	5	1.8	2.1E-01	2	1.1	7.1E-01
PLATZ (11)	0	0.0	1.0E+00	3	5.1	3.1E-02	0	0.0	1.0E+00	1	4.1	2.3E-01
Pseudo (5)	0	0.0	1.0E+00	3	11.2	6.0E-03	0	0.0	1.0E+00	1	9.1	1.2E-01
SBP (16)	0	0.0	1.0E+00	2	2.3	2.3E-01	1	1.9	4.3E-01	0	0.0	1.0E+00
SWI/SNF-BAF60b (16)	0	0.0	1.0E+00	1	1.2	5.9E-01	0	0.0	1.0E+00	2	5.7	5.6E-02
SWI/SNF-SWI3 (5)	0	0.0	1.0E+00	2	7.4	4.6E-02	1	5.9	1.8E-01	1	9.1	1.2E-01
TAZ (7)	1	3.2	2.9E-01	2	5.3	7.4E-02	0	0.0	1.0E+00	0	0.0	1.0E+00
TCP (24)	4	3.8	2.8E-02	0	0.0	6.3E-01	3	3.7	5.6E-02	0	0.0	1.0E+00
TRAF (25)	1	0.9	1.0E+00	2	1.5	6.5E-01	0	0.0	1.0E+00	0	0.0	1.0E+00
TRIHELIX (26)	2	1.7	3.3E-01	2	1.4	6.5E-01	1	1.1	5.9E-01	1	1.7	4.5E-01
WRKY (74)	1	0.3	3.7E-01	0	0.0	3.1E-02	3	1.2	7.4E-01	1	0.6	1.0E+00
zf-AN1 (7)	0	0.0	1.0E+00	2	5.3	7.4E-02	0	0.0	1.0E+00	1	6.5	1.6E-01
zf-HD (17)	4	5.3	1.0E-02	0	0.0	1.0E+00	0	0.0	1.0E+00	0	0.0	1.0E+00
ZIM (15)	3	4.5	3.8E-02	0	0.0	1.0E+00	2	4.0	1.0E-01	0	0.0	1.0E+00

References:

1. Blair, E.J.; Bonnot, T.; Hummel, M.; Hay, E.; Marzolino, J.M.; Quijada, I.A.; Nagel, D.H. Contribution of time of day and the circadian clock to the heat stress responsive transcriptome in Arabidopsis. *Sci. Rep.* **2019**, *9*.
2. Li, B.; Gao, Z.; Liu, X.; Sun, D.; Tang, W. Transcriptional Profiling Reveals a Time-of-Day-Specific Role of REVEILLE 4/8 in Regulating the First Wave of Heat Shock-Induced Gene Expression in Arabidopsis. *Plant Cell* **2019**, *31*, 2353–2369.
3. Mockler, T.C.; Michael, T.P.; Priest, H.D.; Shen, R.; Sullivan, C.M.; Givan, S.A.; McEntee, C.; Kay, S.A.; Chory, J. The DIURNAL project: DIURNAL and circadian expression profiling, model-based pattern matching, and promoter analysis. *Cold Spring Harb. Symp. Quant. Biol.* **2007,** 72, 353–363.

4. Pruneda-Paz, J.L.; Breton, G.; Nagel, D.H.; Kang, S.E.; Bonaldi, K.; Doherty, C.J.; Ravelo, S.; Galli, M.; Ecker, J.R.; Kay, S.A. A genome-scale resource for the functional characterization of Arabidopsis transcription factors. *Cell Rep*. **2014,** 8, 622–632.
